# Multi-modal Analysis of Courtship Behaviour in the Old World Leishmaniasis Vector *Phlebotomus argentipes*

**DOI:** 10.1371/journal.pntd.0003316

**Published:** 2014-12-04

**Authors:** Daniel P. Bray, Khatijah Yaman, Beryl A. Underhilll, Fraser Mitchell, Victoria Carter, James G. C. Hamilton

**Affiliations:** 1 Chemical Ecology Group, Centre for Applied Entomology and Parasitology, Keele University, Keele, United Kingdom; 2 Disease Vector Group, Department of Plant Protection Biology, Swedish University of Agricultural Sciences, Alnarp, Sweden; 3 Entomology and Parasitology Unit, Department of Paraclinical Sciences, Faculty of Medicine and Health Sciences, Universiti Malaysia Sarawak, Kota Samarahan, Malaysia; Fundaçao Oswaldo Cruz, Brazil

## Abstract

**Background:**

The sand fly *Phlebotomus argentipes* is arguably the most important vector of leishmaniasis worldwide. As there is no vaccine against the parasites that cause leishmaniasis, disease prevention focuses on control of the insect vector. Understanding reproductive behaviour will be essential to controlling populations of *P. argentipes*, and developing new strategies for reducing leishmaniasis transmission. Through statistical analysis of male-female interactions, this study provides a detailed description of *P. argentipes* courtship, and behaviours critical to mating success are highlighted. The potential for a role of cuticular hydrocarbons in *P. argentipes* courtship is also investigated, by comparing chemicals extracted from the surface of male and female flies.

**Principal Findings:**

*P. argentipes* courtship shared many similarities with that of both *Phlebotomus papatasi* and the New World leishmaniasis vector *Lutzomyia longipalpis*. Male wing-flapping while approaching the female during courtship predicted mating success, and touching between males and females was a common and frequent occurrence. Both sexes were able to reject a potential partner. Significant differences were found in the profile of chemicals extracted from the surface of males and females. Results of GC analysis indicate that female extracts contained a number of peaks with relatively short retention times not present in males. Extracts from males had higher peaks for chemicals with relatively long retention times.

**Conclusions:**

The importance of male approach flapping suggests that production of audio signals through wing beating, or dispersal of sex pheromones, are important to mating in this species. Frequent touching as a means of communication, and the differences in the chemical profiles extracted from males and females, may also indicate a role for cuticular hydrocarbons in *P. argentipes* courtship. Comparing characteristics of successful and unsuccessful mates could aid in identifying the modality of signals involved in *P. argentipes* courtship, and their potential for use in developing new strategies for vector control.

## Author Summary

The sand fly *Phlebotomus argentipes* transmits *Leishmania* parasites through female blood-feeding. These parasites cause leishmaniasis, a potentially fatal disease for which there is no vaccine. Understanding how insect vectors behave can aid in developing strategies to reduce disease transmission. Here, we investigate courtship behaviour in *P. argentipes*. Courtship is critical to an organism's life cycle, as it is essential for mating and reproduction. We show that courtship in this species begins with the male wing-flapping while approaching the female. This behaviour may suggest production of audio signals, or dispersal of chemicals from the male, which the female finds attractive. There then follows a period of touching between males and females prior to copulation. This behaviour may function in the transmission and reception of chemical signals, present on the insect surface. Many insects use these kinds of chemicals in courtship, and here we show differences in the chemicals extracted from the cuticle of male and female *P. argentipes*. Both males and females were found to be able to reject a potential mate. Understanding why some *P. argentipes* are more attractive than others could help identify the signals essential to reproduction, and their potential for use in vector control.

## Introduction

Visceral leishmaniasis (VL) is a debilitating disease estimated to cause 20,000–40,000 deaths worldwide each year [1]. The Indian subcontinent is one of the areas most affected by VL, with over 140,000 cases per year estimated to occur in India alone [1]. The etiologic agent in this region is the protozoan parasite *Leishmania donovani* (Kinetoplastida: Trypanosomatidae), with the sand fly *Phlebotomus argentipes* (Diptera: Psychodidae) the proven or suspected vector in Bangladesh, India, Nepal and Sri Lanka [2]. As there is no vaccine against VL, and cost and drug resistance limit effectiveness of treatment in India [3], control of the sand fly vector remains a priority for reducing transmission [4]. To be successful these programmes require a thorough understanding of the behaviour of the insect vector [5], not least because many human activities can significantly alter sand fly behaviour and potential risk of transmission. Agricultural practices, for example, may lead to creation of new habitats for sand flies [6]. Insecticide spraying for control can lead to unintentional diversion of sand flies away from normal resting sites in animal houses, potentially increasing the biting risk to humans [7], [8].

Studies of insect vector mating behaviour facilitate development of novel tools for control. For example, a new approach for controlling the South American vector of VL, *Lutzomyia longipalpis*, exploits attraction to male-produced sex pheromones. A synthetic version of this chemical attracts both females and males to traps and insecticide-sprayed surfaces for up to 3 months in the field [8], [9]. Field and laboratory observations suggest that *P. argentipes* shares some underlying behavioural characteristics with *L. longipalpis*. In both species, males form aggregations on or above host animals prior to the arrival of females, where mating and blood-feeding takes place [10], [11]. Currently, very little is known about the signals which mediate male-female interactions in *P. argentipes*. Insect courtship is often a complex process, and can include transmission and reception of auditory, physical, visual and chemical signals between potential mates [12]. In common with *L. longipalpis*, aggregating male *P. argentipes* perform wing-flapping behaviours, but their relevance to mating or courtship is unknown [10], [13]–[16]. There is also evidence that female *P. argentipes* investigate unidentified chemicals that can be extracted from male *P. argentipes* [13]. Hydrocarbons present in the cuticular wax, which function as chemical signals in mating behaviour of many insect species [17], have also been reported from many species of sand fly, including female *P. argentipes* [18]. However, the extent to which male and female *P. argentipes* differ in the hydrocarbons they produce, and how these potential chemicals signals might be transmitted during courtship (e.g. through touching [12]), remains to be investigated.

To date, studies of mating behaviour in *P. argentipes* have been limited to observations of aggregations on host animals [10], [13], [14]. The small-scale interactions between individual males and females, which occur prior to copulation, have not been described. The aim of this study was therefore to provide a detailed analysis of the individual behaviours performed by male and female *P. argentipes* during courtship, and the sequence in which they occur. Behaviours which predicted copulation success, and are therefore critical to mating, were identified through statistical analysis. Courtship in *P. argentipes* was then compared with that of *L. longipalpis* [16] and *Phlebotomus papatasi* [19], species from which there is also evidence of chemical communication [20]. Through a combination of gas chromatography and mathematical analysis, we also determined whether there are sex-specific differences in the chemicals present in or on the male and female cuticle of unmated *P. argentipes*. Such chemicals might play a crucial role in sexual signalling of this important disease vector.

## Methods

### Sand fly rearing

*P. argentipes* were from a colony maintained at Keele University, UK, for approximately 28 generations. Adults were kept in Barraud cages at 27°C, 95% RH, under a 12∶12 light∶dark photocycle. Females were blood fed 3 days post-emergence in accordance with UK Home Office Licence requirements (see Ethical Statement). Male and female *P. argentipes* used in both mating trials and chemical analyses were placed into single-sex cages within 5 h of eclosion (prior to rotation of male genitalia) to prevent mating prior to experiments, and fed only on saturated sugar solution.

### Recording of courtship behaviour

Courtship interactions between 38 pairs of male and female *P. argentipes* were recorded under white florescent light in a purpose built bioassay room at Keele University. The males and females used were between 4 and 6 days old as this is the age at which they are believed to be sexually mature. The room was maintained at 27°C±2°C and 85% rh, with all recordings made between 1400 and 1800 hours. Courtship took place in a round plastic mating arena (22 mm ID×15 mm H) (Figure S1 and S2). The top of the arena was covered with a glass slide (76×26×1 mm) which prevented flies escaping while enabling videoing of courtship behaviour. Recordings were made using a colour video camera (TK-1280E; JVC, London, UK) fitted with a zoom lens (Computar 18–108 mm, f 2.5 manual focus; CBC (Europe) Ltd, London, UK) and supported 30 cm above the courtship arena using a copy stand (CS-920; Tracksys Ltd, Nottingham, UK). Output from the camera was fed through a vertical interval time code (VITC) generator (AEC-BOX-18; Adrienne Electronic Corp., Las Vegas, NV, USA) to a time-lapse security video recorder (VCR) (HS1024; Mitsubishi Electric, Hatfield, UK) set to non-stop recording. A feed from the VCR was sent to a colour monitor (Trinitron KV-14MIU; Sony, Thatcham, UK) to enable camera adjustments and observations while filming. Additional illumination for recording was provided by a fibre optic light source (KL 500; Schott UK Ltd, Stafford, UK).

For each observation, a male fly was placed into the arena, via a round hole made in the side, using a mouth aspirator. After a period of 5 min, the VCR was set to record and a female was placed into the arena using the aspirator. Males were placed into the arena first to mimic the natural behaviour of *P. argentipes*, in which males aggregate on host animals prior to the arrival of females [10]. Each observation was recorded for a maximum of ten minutes, or terminated earlier once the pair had disengaged from copulation. The copulation arena was cleaned with hexane to remove any contaminating volatiles (VWR International Ltd, Leighton Buzzard, UK) and left in a fume hood for the hexane to evaporate prior to reuse. The glass slide was washed with 5% Teepol detergent (VWR International, Lutterworth, United Kingdom), distilled water and acetone (Sigma Aldrich, Gillingham, UK) between trials.

### Analysis of courtship behaviour

Recordings of courtship behaviours were analysed using a PC fitted with a PC-VITC card (Adrienne Electronic Corp., Henderson, USA), running the Observer Base Package for DOS (Version 3.0) and Support Package for Video Tape Analysis (Version 3.1; Noldus Information technology, Wageningen, the Netherlands). Videos of courtship were replayed on the VCR, with the output sent simultaneously to the PC-VITC card and the Sony TV monitor. Behaviour of both male and female *P. argentipes* was coded into mutually exclusive categories (in which only one of the behaviours listed in Table 1 could be performed by each fly at any given time) and entered into the Observer software via a sequence of key presses during video playback. Video images were replayed in slow motion, with key presses in Observer synchronised to the time code recorded onto the video by the VITC generator, as read by the PC-VITC card.

**Table 1:** Behaviours performed during *P. argentipes* courtship.

	Name of behaviour	Description
	**Male & female behaviours**	
1	Not courting	Sand fly remains stationary or moves around the arena without wing-flapping, facing or touching its courtship partner.
2	Stationary wing-flapping	Sand fly remains stationary and flaps both wings simultaneously. Flapping followed a pattern of small vibrations through a slight rotation of the wings followed by a large flap, in which both wings extended to an angle of 45–70° from the body.
3	Touching	Sand fly makes contact with its partner by touching with the tips of the legs or antennae. Contact was most often made with the partner's legs or antennae, and occasionally the abdomen.
4	Facing	Male and female remaining motionless while facing one another.
5	Dipping	Sand fly moves vertically by dipping its abdomen to touch the floor of the arena, often in a repeating pattern.
6	Circling and dipping	Sand fly positions its head towards the arena floor and dips the end of its abdomen while moving in a circle or semi-circle around the same spot. Movement occurred in both clockwise and anticlockwise directions.
7	Copulation	Male and female copulate with the tips of the abdomen joined and facing in opposite directions. Males often flapped their wings until female appeared to accept copulation. Females normally remained motionless but occasionally struggled during copulation.
	**Male-only behaviours**	
8	Abdomen bending	Male bends his abdomen laterally; swinging his terminalia to the left and right, often while female is nearby.
9	Approach-flapping	Male rigorously flaps his wings and steps towards female in an alternating repeating pattern.
10	Copulation attempt	From a position parallel to the female, male bends his abdomen in an attempt to make contact with the female genitalia, often while wing-flapping.

Raw data on the order and duration of behaviours performed during courtship were exported from Observer into R version 3.1 [21]. These data formed the basis of subsequent analysis of behavioural transitions (see below). Frequency or duration of behaviours performed by males and females were compared statistically using Wilcoxon signed rank tests. Fisher's exact test was used to establish which male and female behaviours occurred more frequently in successful and unsuccessful courtships, in order to identify behaviours which predicted mating success.

### Analysis of behavioural transitions

**Figure 1:**
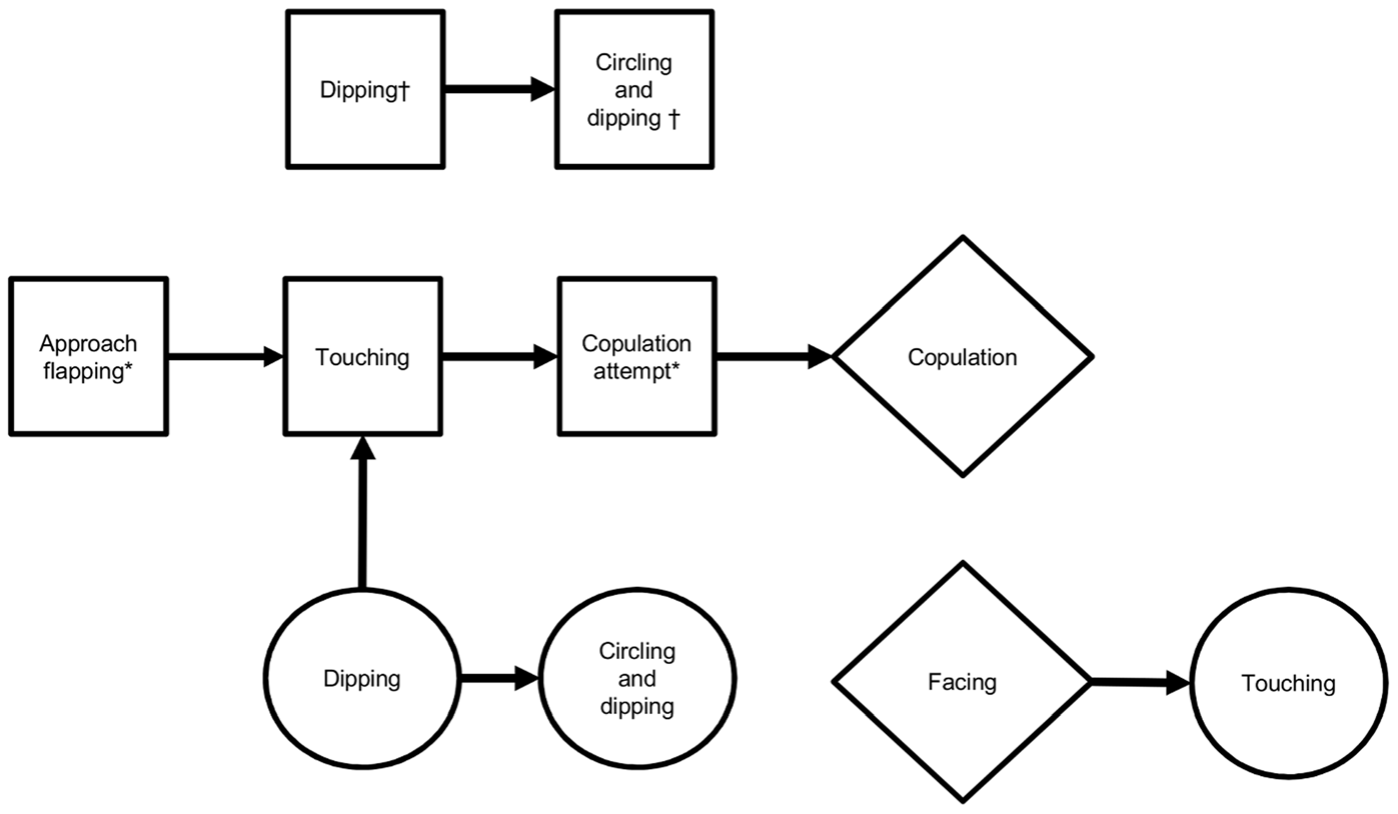
Kinetogram depicting sequence of male (square), female (circle) and joint (diamond) behaviours during *P. argentipes* courtship, based on observation of 38 male-female pairs. * Behaviour significantly (P<0.05) more likely to occur in successful courtships, ending in copulation. † Behaviour significantly more likely to occur in unsuccessful courtships (no copulation).

A log-linear modelling approach in R was used to devise a statistical model of courtship behaviour in *P. argentipes* [16], [19], [22], [23]. Chi-square tests first established whether there was a significant overall association between preceding and following behaviour in male-male, male-female, female-female and female-male behavioural transitions during courtship (Tables S1, S2, S3, S4), ignoring periods of not courting (Table 1, behaviour 1). To improve robustness of **X** ^2^ tests, behaviours which occurred less than five times in rows or columns of transition tables were excluded from analysis. Adjusted residuals >1.96 in a no-effect model identified individual behavioural transitions which occurred significantly more likely than expected by chance in each table [24], [25]. Significant transitions were joined together to form a kinetogram outlining the overall sequence of behaviours in *P. argentipes* courtship (Figure 1).

### Chemical analysis of cuticular profiles

Volatile and non-volatile chemicals present on the surface of the cuticle or in glandular tissue of sexually mature (4–6 day old), unmated male and female *P. argentipes* were extracted by placing individual flies in glass vials containing 20 µl of hexane for 15 minutes [26]. Following removal of flies, vials were sealed and stored at −20°C until use. For gas chromatography–mass spectrometry (GC/MS) analysis, individual extracts were reduced to dryness at room temperature under nitrogen and then re-suspended in 2 µl hexane prior to injection. Samples were analysed via splitless injection (inlet temperature: 280°C) into an Agilent 7890A-5975C GC/MS (Agilent Technologies UK Ltd, Cheshire, UK) on a non-polar HP-5MS column. Oven temperature was maintained at 75°C for 5 min, before rising at 17°C min^−1^ and held at 310°C for 10 min. The carrier gas was hydrogen.

Gas chromatographs expressed as detector response over time from 24 male and 24 female sand flies were imported into R for analysis [27]. Chromatographs were aligned, and variation in baseline was removed using the ptw (parametric time warping) package [28]. Noise in chromatograms was reduced by averaging responses over 25 ms, and ignoring peaks below a threshold of 50000 in height. This resulted in a set of 39 peaks not present in control ‘blank’ samples for further analysis. Principle component analysis (Psych package [29]) was then used to extract and rotate components explaining underlying variation in the matrix of peak heights for the 48 flies. Linear discriminant analysis with jack-knifed predictions (Mass package [30]) was then used to determine the accuracy with which fly sex could be predicted from the scores assigned to each sand fly from the extracted components.

### Ethics statement

Female *P. argentipes* were blood fed on anaesthetized laboratory mice. All work involving blood-feeding was carried out in the UK under UK Home Office licence 4003279 and was approved by the Home Office. The Keele University animal welfare ethics review board at Keele University also reviewed and approved the blood feeding protocol prior to commencement of this study. The study was conducted according to the guidelines set for animal husbandry by Keele University and the UK Home Office. These rules are governed by the Animals (Scientific Procedures) Act 1986. In addition we comply with the Common Rules for Animal Research that are prepared by the UK National Centre for the Replacement, Refinement and Reduction of Animals in Research (NC3Rs).

## Results

### Overview of courtship behaviour

Both male and female *P. argentipes* actively participated in courtship, performing stationary wing-flapping, touching, facing, dipping, and circling and dipping behaviours (Table 1). Males performed three behaviours not performed by females: abdomen bending (Table 1, behaviour 8), approaching the female while wing-flapping (behaviour 9) and attempting copulation (behaviour 10). Bouts of active courtship were separated by periods of not courting (behaviour 1), in which sand flies were either stationary or moving around the arena. In total, males spent a greater proportion of time during trials actively courting than females (median (25%–75% quartiles), males 20.5% (8.7%–35.1%), females 6.6% (3.0–20.5%), Wilcoxon signed rank test *P*<0.01).

While both sexes performed stationary wing-flapping (behaviour 2), males spent more time wing-flapping per trial than females (males: 27.8 s (8.2–90.6), females: 2.4 s (0.0–29.3), *P*<0.001), and wing-flapped more frequently (median behaviours per trial, males: 9.50 (3.0–17.6), females: 2.0 (0.0–10.0), *P*<0.01).

However, there was no difference between sexes in time spent touching per trial (behaviour 3) (males: 2.3 (0.2–6.5), females: 4.0 (0.0–7.6), not significant (NS)), or frequency of touching behaviours initiated per trial (males: 3.0 (1.0–5.8), females: 2.0 (0.0–3.0), NS). Similarly, there was no difference between sexes in time spent dipping (behaviour 5) (males: 0.0 (0.0–4.0), females (0.26 (0.0–8.1), NS) or overall frequency of dipping behaviours (males: 0.0 (0.0–1.0), females: 0.5 (0.0–2.0), NS). There was also no difference in time spent circling and dipping (behaviour 6) (males: 0.0 (0.0–0.0) [mean 3.1 s], females: 0.0 (0.0–0.0) [mean 4.7 s], NS), or frequency of circling and dipping (males 0.0 (0.0–0.0) [mean 0.3 behaviours per trial], females 0.0 (0.0–0.0) [mean 0.3], NS).

Pairs of sand flies spent a median of 2.5 s (0.6–3.7) facing (behaviour 4) in 15 of 38 trials in which this behaviour occurred. Males spent 2.3 s (0.76–4.0) approach flapping (behaviour 8), 1.8 s (1.2–5.2) abdomen bending (behaviour 9) and 0.7 s (0.3–1.7) attempting copulation (behaviour 10), where each of these behaviours occurred during courtship trials.

Courtship proceeded to copulation in 16/38 (42%) of the 10 minute trials. Where copulation occurred, median copulation latency (measured from the beginning of the trial) was 104.1 s (63.6–142.3). In ten cases, copulation was concluded within the 10 min trial, with a median duration of 264.4 s (81.4–315.4). Successful males copulated on their first (8 males) second (6 males) third (one male) or fifth (one male) attempt. Males were observed to continue wing-flapping during 4/16 (25%) copulations. In general, males flapped their wings rapidly when beginning copulation, but then ceased.

### Sequence of behaviours during courtship

An overall effect of preceding behaviour on following behaviour was found in male-male behavioural transitions (**X** ^2^ = 168.7, df = 48, *P*<0.001; Table S1). Significant individual transitions occurred between approach flapping and touching, touching and copulation attempt, and copulation attempt to copulation. A significant transition also occurred between dipping to circling and dipping. Similarly, an effect of preceding behaviour on following behaviour was also found for female-female transitions (**X** ^2^ = 45.5, df = 11, *P*<0.001; Table S2). As for males, a significant transition occurred between dipping and circling and dipping. In addition, there was also a significant transition between facing and touching.

Examining behavioural interactions between sexes, an overall effect of preceding behaviour on following behaviour was found in male to female transitions (**X** ^2^ = 79.9, df = 20, *P*<0.001; Table S3). Male copulation attempt led to copulation, and facing to female touching. An overall effect of preceding behaviour on following behaviour was also found in female to male transitions (**X** ^2^ = 34.3, df = 20, *P*<0.05; Table S4), with the only significant individual transition occurring between female dipping and male touching.

### Behaviours predicting copulation

Two male behaviours, approach flapping (8) and attempting copulation (10) occurred significantly more frequently in courtships leading to copulation, and therefore predicted courtship success (Fisher's exact test, *P*<0.05, Table 2). Two further male behaviours, dipping (5) and circling and dipping (6) occurred more frequently in unsuccessful courtships than successful courtships. These behaviours may therefore signal rejection of the female as a potential mate. Occurrence of individual female behaviours or facing during courtship did not predict copulation (Fisher's exact test, *P*<0.05 Table 2).

**Table 2:** Behaviours predicting copulation during *P. argentipes* courtships.

	Unsuccessful courtships (n = 22)†	Successful courtships (n = 16)‡
**Male behaviours**		
Approach flapping	36.4%	81.3%**
Copulation attempt	9.1%	93.8%***
Abdomen bending	18.2%	50.0%
Circling and dipping	27.3%*	0.0%
Dipping	45.5%*	12.5%
Stationary wing-flapping	95.5%	100.0%
Touching	81.8%	87.5%
**Female behaviours**		
Circling and dipping	31.8%	6.3%
Dipping	59.1%	37.5%
Stationary wing-flapping	68.2%	62.5%
Touching	86.4%	56.3%
**Joint behaviours**		
Facing	45.5%	31.3%

† Percentage of unsuccessful courtships (no copulation) in which the behaviour occurs.

‡ Percentage of successful courtships (copulation) in which the behaviour occurs. Asterisks indicate behaviours which occurred significantly more frequently in unsuccessful or successful courtships (Fishers exact test on count data: * P<0.05, ** P<0.01, *** P<0.001).

### Kinetogram of courtship behaviour

Analysis of behavioural transitions and occurrence of behaviours in successful and unsuccessful copulations suggests the following model of courtship in *P. argentipes* (Figure 1). In successful copulations, the male progresses from approach flapping, to touching, to attempting copulation, and copulation (Video S1). Dipping and circling and dipping appear to be related behaviours, and may indicate an unwillingness to mate. Female dipping was found to lead to the male touching the female, while periods of facing were followed by female touching the male. Both may indicate an attempt to investigate or prompt an unwilling mate.

### Analysis of cuticular extracts

**Figure 2:**
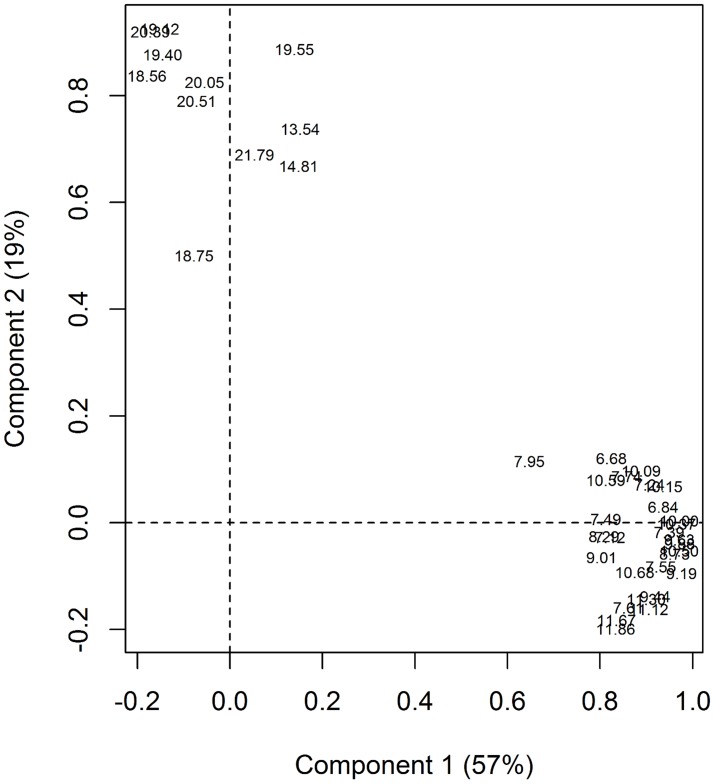
Component loadings for gas chromatogram peaks with different retention times extracted from 24 male and 24 female *P. argentipes*. Peaks with lower retention times had higher loadings for rotated component 1, which explained 57% of the variation in the original dataset. Peaks with higher retention times had higher loading for component 2, which explained 19% of the original variation.

Two varimax-rotated principle components were extracted from the matrix of 39 peak heights derived from male and female *P. argentipes*. These two components explained 57% and 19% of the variation in peak height respectively. Plotting of component loading indicated that component 1 scaled positively with peaks with relatively short retention times (6.68–11.86 minutes; Figure 2, x axis). Component 2, scaled with peaks with relatively long retention times (13.54–21.79 minutes; Figure 2, y axis).

**Figure 3:**
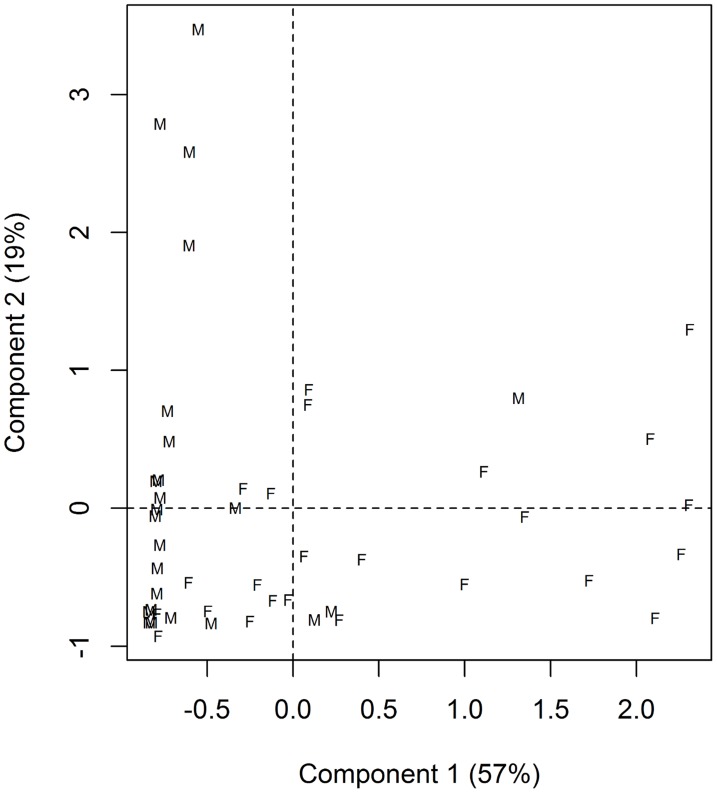
PCA component scores for 24 male and 24 female *P. argentipes*. Females had generally higher scores for component 1, while males showed greater variation in scores along component 2.

**Figure 4:**
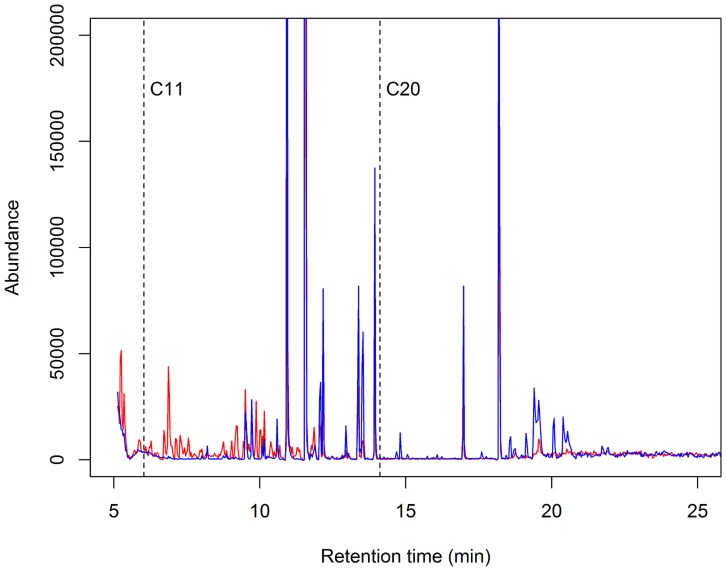
Example cleaned gas chromatographs extracted from individual male (blue line) and female (red line) *P. argentipes*. Females appeared to possess chemicals with lower retention times (less than 12 minutes) not recovered from males. Conversely males had larger peaks for chemicals present at retention times greater than 18 minutes. Dotted vertical lines represent retention times for undecane (C11) and eicosane (C20) under the same temperature programme.

Plotting component scores for individual *P. argentipes*, females had higher scores for component 1, while males showed greater variation on component 2 (Figure 3). This translates to female extracts exhibiting higher peaks for chemicals with shorter retention times (which may not be present in males), and males higher peaks for chemicals with longer retention times (Figure 4).

Linear discriminate function analysis performed on the two rotated components resulted in jack-knife predictions of fly sex (male or female) which were significantly better than chance (fly sex correctly predicted in 75% of cases, Fishers exact test, *P*<0.001). Predictions for males (21/24, 88% of individuals correctly sexed) were more accurate than those for females (15/24, 63%). This difference in predictive ability may reflect the general absence of variation in males in component 1: i.e. peaks with low retention times present in females, but not males (Figure 4).

## Discussion

Courtship behaviour in *P. argentipes* shared several similarities with both *P. papatasi* and the new world leishmaniasis vector *L. longipalpis*. The core progression of behaviours comprised the male wing-flapping while approaching the female, before touching her with the legs or antennae prior to attempting copulation. This builds on a previous description of the ‘courtship dance’ of *P. argentipes*, described as involving males hopping, swinging the terminalia and wing-flapping [14]. Both female and male *P. argentipes* engaged in wing-flapping behaviour during courtship, with male approach flapping a significant predictor of copulation success. While integral to *P. argentipes* courtship, the function of wing-flapping in this species is currently unknown. In *L. longipalpis*, male wing-flapping has been hypothesised to aid in dispersal of attractive sex pheromones released from abdominal tergites [15], [31]. These pheromones attract female *L. longipalpis* to aggregations of males formed on or above host animals [32]. Male *P. argentipes* also form mating aggregations on cows or other animals, and perform wing-flapping behaviours prior to the arrival of females [10], [13]. It is therefore possible that male *P. argentipes* also release an attractive sex pheromone to aid females in locating these aggregations. Male *P. argentipes* also performed abdomen bending during courtship, a behaviour previously reported from *Phlebotomus papatasi* [19], *Phlebotomus longipes* [33] *Phlebotomus martini* [34] and *Lutzomyia vexator* [35]. This could conceivably also function in pheromone release from abdominal tergites, the site of production of pheromones in *L. longipalpis*, [36]. There is behavioural evidence of chemically mediated attraction of females to males in both *P. argentipes* and *P. papatasi* [13], [20]. However, to date no sex pheromone, or likely sex pheromone-producing structure, has been identified in any of the abdomen-bending sand flies [37], and *L. longipalpi*s (which does produce pheromones) does not perform this behaviour [16].

In addition to chemical communication, *P. argentipes* wing-flapping may also function in production of audio signals important to mating. Courtship songs, produced by rhythmic wing vibrations are believed to play a role in species recognition in *L. longipalpis*, as the pattern of sound produced by males during copulation differs between members of the species complex [38], [39]. Similar audio signals have also been recorded during courtship in *Lutzomyia intermedia* [40], and during copulation in *Lutzomyia cruzi* [41] and *Lutzomyia migonei* [42]. To our knowledge, no audio signals have been recorded from *P. argentipes*, despite descriptions of wing-flapping in this and other Old World species [19], [43]. As in *P. papatasi*, male *P. argentipes* flapped their wings only briefly at the start of copulation [19], possibly to assist in alignment of the male and female genitalia. This may suggest that audio signals produced prior to copulation (rather than during) may play a greater role in courtship. Manipulative playback experiments, similar to those carried out in *Drosophila* [44] are needed to determine the function of audio signals (if any) in sand fly mating behaviour.

Whether associated with chemical, audio or visual signals, wing-flapping appears to be a predominantly male activity, with male *P. argentipes* wing-flapping more frequently, and for longer periods of time than females. The same trend has previously been observed in both *P. papatasi* and *L. longipalpis* [16], [19]. In these species, female wing-flapping was found to be a predictor of courtship success, possibly indicating a willingness to mate. The same was not found to be the case of *P. argentipes* reported here.

Touching, initiated by both male and female *P. argentipes*, was frequently observed during courtship. This behaviour has also been reported from studies of *P. papatasi* and *L. longipalpis* [16], [19]. Whilst found to be an integral part of the behavioural progression towards copulation, occurrence of this behaviour does not in itself predict copulation success in any of the three species examined to date [16], [19]. Touching in many insects, including member of the genus *Drosophila*, is involved in the transmission of short-range pheromones during courtship [12], [45]. These chemicals include cuticular hydrocarbons, which can provide a range of information including species, sex, age and mating status of a potential partner [46]. Here, GC analysis revealed consistent differences in the profile of chemicals extracted from the surface of male and female *P. argentipes*. Comparison of retention times with straight chain alkanes suggest the recovered female-associated chemicals may be smaller than the C20–C40 chemicals normally recovered from cuticle wax [47]. The male-associated peaks however appear to be in the range for cuticular hydrocarbons, although identification will be required to confirm their structure. Previous studies have revealed variation in similar extracts from female *P. argentipes* from different regions, and between wild and colonized females [18]. Taken together, the results here indicate that there are also differences in the chemical profile of males and females, and that a potential behavioural mechanism exists for transmission and reception of these chemicals (touching). However, this is not in itself evidence for sex pheromones: more work is required to identify the potential chemicals involved, and to conduct bioassays to ascertain their relevance to mating and other behaviour. In particular, experiments are needed to determine whether the male-associated chemicals detected here could be responsible for the response of female *P. argentipes* to male extracts [13].

Courtship analysis revealed that male *P. argentipes* could signal an unwillingness to mate by dipping their abdomen toward the surface of the arena. When this occurred, copulation was significant less likely to occur. Similar abdomen dipping behaviour has previously been observed in female *L. longipalpis*, which are free to choose from a number of potential mates within a lek [15]. It has been suggested that in *L. longipalpis* this behaviour is linked to monandry as for the female the correct mate choice is essential. Why male *P. argentipes* should reject a potential mate is unclear, as males make relatively little contribution to offspring production. As only virgin males were used in this study, sperm depletion is also unlikely to explain this result. Further work is needed to ascertain whether rejection of females is a genuine feature of mating behaviour of *P. argentipes*, or an artefact of the trial conditions. If chemically mediated, mate rejection could form a target for mating disruption as a means of vector control.

Where mating did take place, *P. argentipes* copulated back to back, as occurs in most species of sand fly. There was no evidence of piggy backing behaviour (a possible mate-guarding activity), as performed by *Phlebotomus duboscqi* [48]. As in *L. longipalpis* and *P. papatasi*, there was considerable variation in both copulation latency, and the duration of copulation in *P. argentipes* [16], [19]. The extent to which the latter is related to successful transfer of sperm and subsequent fertilization is unknown.

Very little is known about the mating strategy of *P. argentipes*. Experiments to answer questions such as whether females mate only once or more often, or why males appear to reject females are essential for developing control strategies. The results of this study demonstrate that courtship in *P. argentipes* shares similarities with both the new world VL vector *L. longipalpis*, and the Old World cutaneous leishmaniasis vector *P. papatasi*. As wing-flapping seems crucial to mating in this species, future studies should attempt to identify the modality of the signal produced by this behaviour, and its potential for exploitation as a means of vector control. Similarly, chemical analyses and behavioural bioassays are now required to identify the chemicals present on the surface of male and female *P. argentipes*, and to determine if they have any role in attracting or dissuading potential mates. Both sexes of *P. argentipes* reject potential mates, which suggests that some individuals are more attractive than others. *L. longipalpis* females are known to prefer a small number of males within an aggregation, and attractiveness in this species is both an inheritable characteristic, and associated with pheromone production [15], [49]. Identifying differences between relatively attractive and unattractive individuals in *P. argentipes* would be a logical next step in identifying the modality of sexual signals used in this species, and their potential for exploitation in vector control.

## Supporting Information

Figure S1 Close-up image of the arena used to observe male/female courtship interactions. The image shows the walls of the arena resting on a glass slide covered with a glass coverslip. For each observation, a male fly was placed into the arena, via a round hole made in the side, using a mouth aspirator.
(JPG)

Figure S2 Close-up image of the arena used to observe male/female courtship interactions. The image shows the walls of the arena resting on a glass slide covered with a glass coverslip. For each observation, a male fly was placed into the arena, via a round hole made in the side, using a mouth aspirator.
(JPG)

Table S1 Frequencies of male to male behaviours.
(DOCX)

Table S2 Frequencies of female to female behaviours.
(DOCX)

Table S3 Frequencies of male to female behaviours.
(DOCX)

Table S4 Frequencies of female to male behaviours.
(DOCX)

Video S1 Courtship behaviour of male and female *P. argentipes*. The thinner male, identifiable by the genital clasper at the end of the abdomen, approaches the female while wing-flapping (Table 1, behaviour 9), who also performs wing-flapping while stationary (behaviour 2).The male makes contact with the female through touching with the legs or antennae (behaviour 3) several times prior to copulation.
(MP4)

## References

[1] Alvar J, Vélez ID, Bern C, Herrero M, Desjeux P, et al (2012) Leishmaniasis worldwide and global estimates of its incidence. PLoS ONE 7: e35671 10.1371/journal.pone.0035671 22693548 PMC3365071

[2] Maroli M, Feliciangeli MD, Bichaud L, Charrel RN, Gradoni L (2013) Phlebotomine sandflies and the spreading of leishmaniases and other diseases of public health concern. Med Vet Entomol 27: 123–147 10.1111/j.1365-2915.2012.01034.x 22924419

[3] Sundar S (2001) Drug resistance in Indian visceral leishmaniasis. Trop Med Int Health 6: 849–854 10.1046/j.1365-3156.2001.00778.x 11703838

[4] Davies CR, Kaye P, Croft SL, Sundar S (2003) Leishmaniasis: new approaches to disease control. Br Med J 326: 377–382 10.1136/bmj.326.7385.377 12586674 PMC1125241

[5] Warburg A, Faiman R (2011) Research priorities for the control of phlebotomine sand flies. J Vector Ecol J Soc Vector Ecol 36 Suppl 1: S10–16 10.1111/j.1948-7134.2011.00107.x 21366761

[6] Kesari S, Mandal R, Bhunia GS, Kumar V, Das P (2014) Spatial distribution of *P. argentipes* in association with agricultural surrounding environment in North Bihar, India. J Infect Dev Ctries 8: 358–364 10.3855/jidc.3353 24619268

[7] Kelly DW, Mustafa Z, Dye C (1997) Differential application of lambda-cyhalothrin to control the sandfly *Lutzomyia longipalpis* . Med Vet Entomol 11: 13–24 10.1111/j.1365-2915.1997.tb00285.x 9061673

[8] Bray DP, Alves GB, Dorval ME, Brazil RP, Hamilton JG (2010) Synthetic sex pheromone attracts the leishmaniasis vector *Lutzomyia longipalpis* to experimental chicken sheds treated with insecticide. Parasit Vectors 3: 16 10.1186/1756-3305-3-16 20222954 PMC2850890

[9] Bray DP, Carter V, Alves GB, Brazil RP, Bandi KK, et al (2014) Synthetic sex pheromone in a long-lasting lure attracts the visceral leishmaniasis vector, *Lutzomyia longipalpis*, for up to 12 weeks in Brazil. PLoS Negl Trop Dis 8: e2723 10.1371/journal.pntd.0002723 24651528 PMC3961206

[10] Lane RP, Pile MM, Amerasinghe FP (1990) Anthropophagy and aggregation behaviour of the sandfly *Phlebotomus argentipes* in Sri Lanka. Med Vet Entomol 4: 79–88 10.1111/j.1365-2915.1990.tb00263.x 2132972

[11] Morrison A, Ferro C, Pardo R, Torres M, Wilson M, et al (1995) Nocturnal activity patterns of *Lutzomyia longipalpis* (Diptera, Psychodidae) at an endemic focus of visceral leishmaniasis in Colombia. J Med Entomol 32: 605–617. 7473615 10.1093/jmedent/32.5.605

[12] Greenspan RJ, Ferveur JF (2000) Courtship in Drosophila. Annu Rev Genet 34: 205–232 10.1146/annurev.genet.34.1.205 11092827

[13] Kumar V, Krishnakumari B, Kesari S, Kumari K, Kumar R, et al (2012) Preliminary observations on the female behavior of the Indian sandfly vector, *Phlebotomus argentipes* (Diptera: Psychodidae). Ann Entomol Soc Am 105: 201–205 10.1603/AN11089

[14] Palit A, Kesari S, Ranjan A, Kishore K (1993) Mating aggregation of *Phlebotomus argentipes* at animal hosts in India. Indian J Parasitol 17: 11–13 10.1603/AN11089

[15] Jones TM, Hamilton JGC (1998) A role for pheromones in mate choice in a lekking sandfly. Anim Behav 56: 891–898 10.1006/anbe.1998.0857 9790700

[16] Bray DP, Hamilton JGC (2007) Courtship behaviour in the sandfly *Lutzomyia longipalpis*, the New World vector of visceral leishmaniasis. Med Vet Entomol 21: 332–338 10.1111/j.1365-2915.2007.00700.x 18092971

[17] Howard RW, Blomquist GJ (2005) Ecological, behavioral, and biochemical aspects of insect hydrocarbons. Annu Rev Entomol 50: 371–393 10.1146/annurev.ento.50.071803.130359 15355247

[18] Kamhawi S, Lane RP, Cameron M, Phillips A, Milligan P, et al (1992) The cuticular hydrocarbons of *Phlebotomus argentipes* (Diptera, Phlebotominae) from field populations in northern India and Sri Lanka, and their change with laboratory colonization. Bull Entomol Res 82: 209–212 10.1017/S0007485300051749

[19] Chelbi I, Bray DP, Hamilton JGC (2012) Courtship behaviour of *Phlebotomus papatasi* the sand fly vector of cutaneous leishmaniasis. Parasit Vectors 5: 179 10.1186/1756-3305-5-179 22935092 PMC3480941

[20] Chelbi I, Zhioua E, Hamilton JGC (2011) Behavioral evidence for the presence of a sex pheromone in male *Phlebotomus papatasi* Scopoli (Diptera: Psychodidae). J Med Entomol 48: 518–525 10.1603/ME10132 21661311

[21] R Development Core Team (2012) R: A Language and Environment for Statistical Computing. Vienna, Austria: R Foundation for Statistical Computing. Available: http://www.R-project.org/.

[22] Liimatainen J, Aspi HAJ, Welbergen P (1992) Courtship in *Drosophila montana*: the effects of auditory signals on the behaviour of flies. Anim Behav 43: 35–48 10.1016/S0003-3472(05)80069-7

[23] Welbergen PH, Dijken FR, Scharloo W (1987) Collation of the courtship behaviour of the sympatric species *Drosophila melanogaster* and *Drosophila simulans* . Behaviour 101: 1253–1274 10.1163/156853987X00017

[24] Bakeman R, Adamson LB, Stisik P (1995) Lags and logs: statistical approaches to interaction (SPSS version). The Analysis of Change. pp. 279–308.

[25] Bakeman R, Gottman JM (1997) Observing Interaction: an Introduction to Sequential Analysis. Cambridge: Cambridge University Press.

[26] Phillips A, Milligan PJM, Broomfield G, Molyneux DH (1988) Identification of medically important Diptera by analysis of cuticular hydrocarbons. In: Service MW, editor. Biosystematics of Haematophagous Insects. Oxford: Clarendon Press, Vol. Systematics Association Special Volume 37. pp. 39–59.

[27] Wehrens R (2011) Chemometrics with R: multivariate data analysis in the natural sciences and life sciences. Heidelberg; New York: Springer.

[28] Bloemberg Tom G, Gerretzen Jan, Wouters Hans JP, Gloerich Jolein, van Dael Maurice, et al (2010) Improved parametric time warping for proteomics. Chemom Intell Lab Syst 104: 65–74 10.1016/j.chemolab.2010.04.008

[29] Revelle W (2014) psych: Procedures for Psychological, Psychometric, and Personality Research. Evanston, Illinois: Northwestern University.

[30] Venables WN, Ripley BD, Venables WN (2002) Modern applied statistics with S. New York: Springer.

[31] Boufana B, Ward RD, Phillips A (1986) Development of the tergal pheromone gland in male *Lutzomyia longipalpis* (Diptera: Psychodidae). Trans R Soc Trop Med Hyg 80: 333–333.

[32] Kelly DW, Dye C (1997) Pheromones, kairomones and the aggregation dynamics of the sandfly *Lutzomyia longipalpis* . Anim Behav 53: 721–731 10.1006/anbe.1996.0309

[33] Gemetchu T (1976) The biology of a laboratory colony of *Phlebotomus longipes* Parrot & Martin (Diptera: Phlebotomidae). J Med Entomol 12: 661–671. 1263217 10.1093/jmedent/12.6.661

[34] Beach R, Young DG, Mutinga MJ (1983) New phlebotomine sand fly colonies: rearing *Phlebotomus martini*, *Sergentomyia schwetzi*, and *Sergentomyia africana* (Diptera: Psychodidae). J Med Entomol 20: 579–584. 6644755 10.1093/jmedent/20.6.579

[35] Chaniotis BN (1967) The biology of California Phlebotomus (Diptera: Psychodidae) under laboratory conditions. J Med Entomol 4: 221–233. 6052128 10.1093/jmedent/4.2.221

[36] Hamilton JGC, Dougherty MJ, Ward RD (1994) Sex pheromone activity in a single component of tergal gland extract of *Lutzomyia longipalpis* (Diptera, Psychodidae) from Jacobina, Northeastern Brazil. J Chem Ecol 20: 141–151 10.1007/BF02065997 24241705

[37] Ward R, Hamilton J, Dougherty M, Falcao A, Feliciangeli M, et al (1993) Pheromone disseminating structures in tergites of male phlebotomines (Diptera, Psychodidae). Bull Entomol Res 83: 437–445 10.1017/S0007485300029357

[38] Souza NA, Andrade-Coelho CA, Vigoder FM, Ward RD, Peixoto AA (2008) Reproductive isolation between sympatric and allopatric Brazilian populations of *Lutzomyia longipalpis* s.l. (Diptera: Psychodidae). Mem Inst Oswaldo Cruz 103: 216–219 10.1590/S0074-02762008000200017 18425278

[39] Souza NA, Vigoder FM, Araki AS, Ward RD, Kyriacou CP, et al (2004) Analysis of the copulatory courtship songs of *Lutzomyia longipalpis* in six populations from Brazil. J Med Entomol 41: 906–913 10.1603/0022-2585-41.5.906 15535620

[40] Vigoder FM, Souza NA, Peixoto AA (2011) Acoustic signals in the sand fly *Lutzomyia (Nyssomyia) intermedia* (Diptera: Psychodidae). Parasit Vectors 4: 76 10.1186/1756-3305-4-76 21569534 PMC3114007

[41] Vigoder FM, Araki AS, Bauzer LGSR, Souza NA, Brazil RP, et al (2010) Lovesongs and period gene polymorphisms indicate *Lutzomyia cruzi* (Mangabeira, 1938) as a sibling species of the *Lutzomyia longipalpis* (Lutz and Neiva, 1912) complex. Infect Genet Evol 10: 734–739 10.1016/j.meegid.2010.05.004 20478408

[42] Vigoder FM, Souza NA, Peixoto AA (2010) Copulatory courtship song in *Lutzomyia migonei* (Diptera: Psychodidae). Mem Inst Oswaldo Cruz 105: 1065–1067 10.1590/S0074-02762010000800020 21225208

[43] Bray D, Ward R, Hamilton J (2010) The chemical ecology of sandflies (Diptera: Psychodidae). In: Takken W, editor. Ecology and Control of Vector-Borne Diseases. Volume 2. Olfaction in Vector-Host Interactions. Wageningen: Wageningen Academic Publishers. pp. 203–216.

[44] Talyn BC, Dowse HB (2004) The role of courtship song in sexual selection and species recognition by female *Drosophila melanogaster* . Anim Behav 68: 1165–1180 10.1016/j.anbehav.2003.11.023

[45] Ferveur JF (2005) Cuticular hydrocarbons: Their evolution and roles in Drosophila pheromonal communication. Behav Genet 35: 279–295 10.1007/s10519-005-3220-5 15864443

[46] Everaerts C, Farine JP, Cobb M, Ferveur JF (2010) Drosophila cuticular hydrocarbons revisited: mating status alters cuticular profiles. PLoS ONE 5: e9607 10.1371/journal.pone.0009607 20231905 PMC2834761

[47] Phillips A, Milligan P (1986) Cuticular hydrocarbons distinguish sibling species of vectors. Parasitol Today 2: 180–181 10.1016/0169-4758(86)90152-3 15462816

[48] Valenta D, Killick-Kendrick R, Killick-Kendrick M (2000) Courtship and mating by the sandfly *Phlebotomus duboscqi*, a vector of zoonotic cutaneous leishmaniasis in the Afrotropical region. Med Vet Entomol 14: 207–212 10.1046/j.1365-2915.2000.00225.x 10872866

[49] Jones TM, Quinnell RJ, Balmford A (1998) Fisherian flies: benefits of female choice in a lekking sandfly. Proc R Soc Lond B Biol Sci 265: 1651–1657 10.1098/rspb.1998.0484

