# Supplementary figures and images for: Multi-modal Analysis of Courtship Behaviour in the Old World Leishmaniasis Vector Phlebotomus argentipes
Source: PLoS Negl Trop Dis. 2014 Dec 4;8(12):e3316. doi: 10.1371/journal.pntd.0003316 (PMC4256473; doi:10.1371/journal.pntd.0003316)

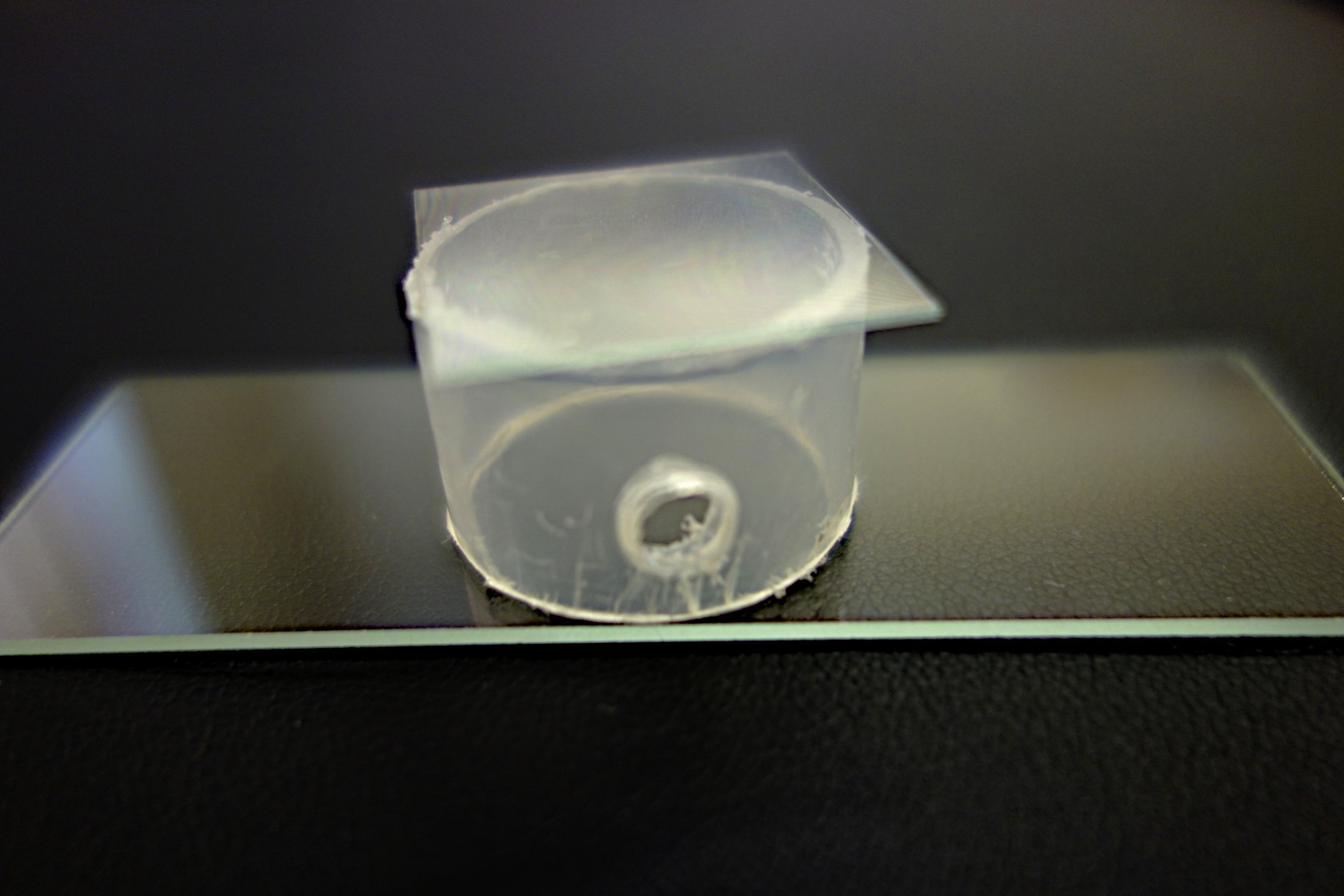

Supplement: Figure S1 — Close-up image of the arena used to observe male/female courtship interactions. The image shows the walls of the arena resting on a glass slide covered with a glass coverslip. For each observation, a male fly was placed into the arena, via a round hole made in the side, using a mouth aspirator. (JPG) [file pntd.0003316.s001.jpg]

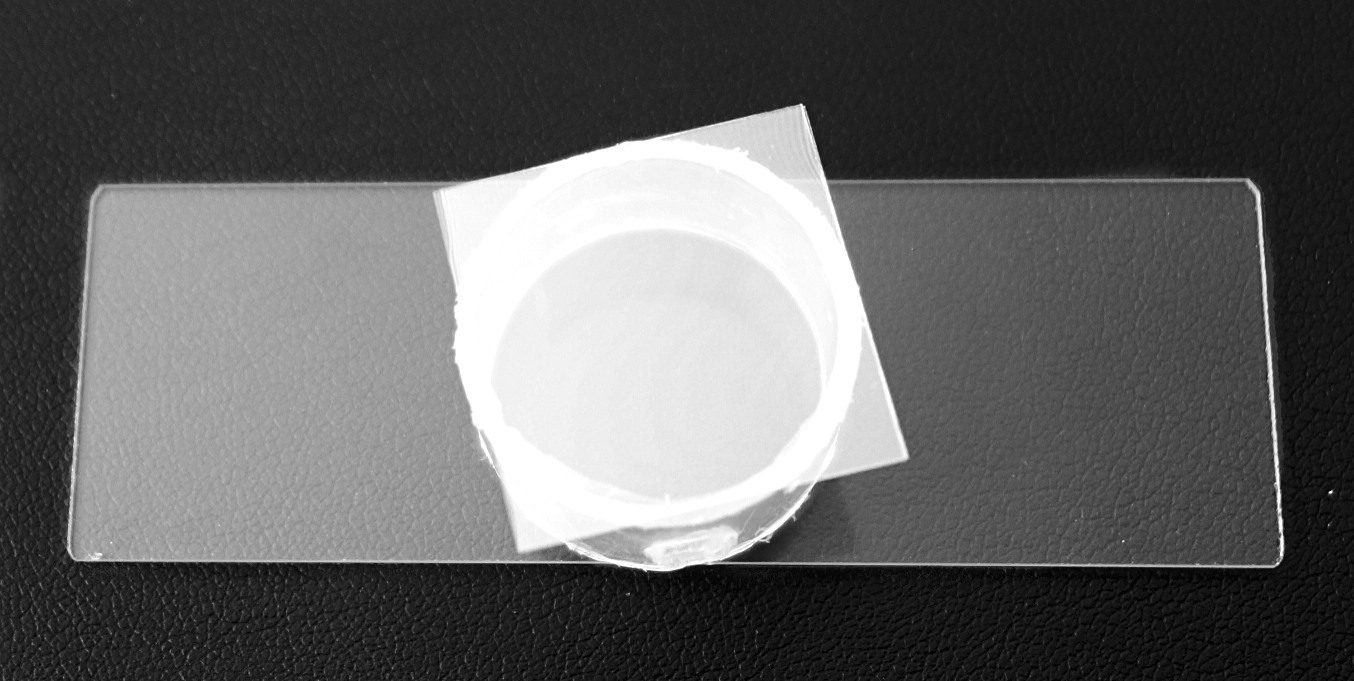

Supplement: Figure S2 — Close-up image of the arena used to observe male/female courtship interactions. The image shows the walls of the arena resting on a glass slide covered with a glass coverslip. For each observation, a male fly was placed into the arena, via a round hole made in the side, using a mouth aspirator. (JPG) [file pntd.0003316.s002.jpg]
