# Supplementary material for: Multi-modal Analysis of Courtship Behaviour in the Old World Leishmaniasis Vector Phlebotomus argentipes
Source: PLoS Negl Trop Dis. 2014 Dec 4;8(12):e3316. doi: 10.1371/journal.pntd.0003316 (PMC4256473; doi:10.1371/journal.pntd.0003316)
Supplement: Table S1 — Frequencies of male to male behaviours. (DOCX) [file pntd.0003316.s003.docx]

**Table S1: Frequencies of male to male behaviours**

|  | **Following behaviour** | |  |  |  |  |  |  |  |
| --- | --- | --- | --- | --- | --- | --- | --- | --- | --- |
| **Preceding** **behaviour** | Abdomen bending | Approach flapping | Circling and dipping | Copulation attempt | Dipping | Facing | Stationary wing-flapping | Touching | Copulation |
| Abdomen bending | - | 4 | 0 | 3 | 1 | 0 | 18 | 2 | 0 |
| Approach flapping | 6 | - | 0 | 7 | 1 | 2 | 12 | 28* | 0 |
| Circling and dipping | 0 | 0 | - | 0 | 1 | 0 | 6 | 1 | 0 |
| Copulation attempt | 4 | 0 | 0 | - | 1 | 0 | 18 | 1 | 9* |
| Dipping | 0 | 1 | 4* | 0 | - | 1 | 17 | 3 | 0 |
| Facing | 0 | 0 | 0 | 0 | 0 | - | 7 | 5 | 0 |
| Stationary wing-flapping | 10 | 37 | 8 | 6 | 20 | 6 | - | 60 | 7 |
| Touching | 6 | 10 | 0 | 15* | 4 | 4 | 78 | - | 0 |

*****Significant positive transition (P<0.05).
