# Supplementary material for: Multi-modal Analysis of Courtship Behaviour in the Old World Leishmaniasis Vector Phlebotomus argentipes
Source: PLoS Negl Trop Dis. 2014 Dec 4;8(12):e3316. doi: 10.1371/journal.pntd.0003316 (PMC4256473; doi:10.1371/journal.pntd.0003316)
Supplement: Table S2 — Frequencies of female to female behaviours. (DOCX) [file pntd.0003316.s004.docx]

**Table S2: Frequencies of female to female behaviours**

|  | **Following behaviour** | | | | | |
| --- | --- | --- | --- | --- | --- | --- |
| **Preceding behaviour** | Circling and dipping | Dipping | Facing | Stationary wing-flapping | Touching | Copulation |
| Circling and dipping | - | 0 | 1 | 6 | 1 | 0† |
| Dipping | 10* | - | 1 | 11 | 6 | 0† |
| Facing | 0 | 0 | - | 3 | 8* | 0† |
| Stationary wing-flapping | 4 | 23 | 7 | - | 26 | 1† |
| Touching | 0 | 6 | 1 | 24 | - | 0† |

*****Significant positive transition (P<0.05)

† Significance of individual transitions not assessed (see text for details).
