# Supplementary material for: Multi-modal Analysis of Courtship Behaviour in the Old World Leishmaniasis Vector Phlebotomus argentipes
Source: PLoS Negl Trop Dis. 2014 Dec 4;8(12):e3316. doi: 10.1371/journal.pntd.0003316 (PMC4256473; doi:10.1371/journal.pntd.0003316)
Supplement: Table S3 — Frequencies of male to female behaviours. (DOCX) [file pntd.0003316.s005.docx]

**Table S3: Frequencies of male to female behaviours**

|  | **Following behaviour** | | | | | |
| --- | --- | --- | --- | --- | --- | --- |
| **Preceding behaviour** | Circling and Dipping | Copulation | Dipping | Facing | Stationary wing-flapping | Touching |
| Abdomen bending | 0† | 0 | 0 | 0 | 3 | 1 |
| Approach flapping | 0† | 0 | 0 | 2 | 6 | 1 |
| Copulation attempt | 0† | 9* | 0 | 0 | 2 | 0 |
| Circling and Dipping | 0† | 0† | 0† | 0† | 3† | 0† |
| Dipping | 0† | 0† | 2† | 1† | 3† | 4† |
| Facing | 0† | 0 | 0 | 1 | 3 | 8* |
| Stationary wing-flapping | 1† | 7 | 14 | 5 | 92 | 27 |
| Touching | 0† | 0 | 6 | 3 | 9 | 5 |

*****Significant positive transition (P<0.05)

† Significance of individual transitions not assessed (see text for details).
