# Supplementary material for: Multi-modal Analysis of Courtship Behaviour in the Old World Leishmaniasis Vector Phlebotomus argentipes
Source: PLoS Negl Trop Dis. 2014 Dec 4;8(12):e3316. doi: 10.1371/journal.pntd.0003316 (PMC4256473; doi:10.1371/journal.pntd.0003316)
Supplement: Table S4 — Frequencies of female to male behaviours. (DOCX) [file pntd.0003316.s006.docx]

**Table S4: Frequencies of female to male behaviours**

|  | **Following behaviour** | | | | | | | | |
| --- | --- | --- | --- | --- | --- | --- | --- | --- | --- |
| **Preceding behaviour** | Approach flapping | Copulation attempt | Abdomen bending | Circling and dipping | Copulation attempt | Dipping | Facing | Stationary wing-flapping | Touching |
| Circling and dipping | 0 | 0† | 0 | 0† | 0† | 0 | 1 | 4 | 0 |
| Dipping | 2 | 0† | 0 | 0† | 0† | 2 | 1 | 10 | 11* |
| Facing | 0 | 0† | 0 | 0† | 0† | 1 | 0 | 6 | 6 |
| Stationary wing-flapping | 7 | 3† | 6 | 0† | 0† | 3 | 7 | 80 | 16 |
| Touching | 1 | 1† | 0 | 0† | 0† | 1 | 1 | 35 | 10 |

*****Significant positive transition (P<0.05)

† Significance of individual transitions not assessed (see text for details).
